# Eating speed and abdominal adiposity in middle-aged adults: a cross-sectional study in Vietnam

**DOI:** 10.1186/s12889-023-15328-0

**Published:** 2023-03-07

**Authors:** Dong Van Hoang, Ami Fukunaga, Chau Que Nguyen, Thuy Thi Phuong Pham, Rachana Manandhar Shrestha, Danh Cong Phan, Huy Xuan Le, Hung Thai Do, Masahiko Hachiya, Tetsuya Mizoue, Yosuke Inoue

**Affiliations:** 1 Department of Epidemiology and Prevention, Center for Clinical Sciences, National Center for Global Health and Medicine, Toyama 1-21-1, Shinjuku- ku, 1628655 Tokyo, Japan; 2 Department of Non-communicable Disease Control and Nutrition, Pasteur Institute in Nha Trang, Khanh Hoa, Vietnam; 3 Pasteur Institute in Nha Trang, Khanh Hoa, Vietnam; 4 Bureau of International Health Cooperation, National Center for Global Health and Medicine, Tokyo, Japan

**Keywords:** Abdominal adiposity, Waist circumference, Waist-to-height ratio, Eating speed, Vietnam

## Abstract

**Background:**

Several studies have associated fast eating speed with the risk of general obesity, but there are inadequate data on the association between eating speed and abdominal adiposity which may pose a higher threat to health than general obesity. The present study aimed to investigate the association between eating speed and abdominal obesity in a Vietnamese population.

**Methods:**

Between June 2019 and June 2020, the baseline survey of an ongoing prospective cohort study on the determinants of cardiovascular disease in Vietnamese adults was conducted. A total of 3,000 people aged 40–60 years old (1,160 men and 1,840 women) were recruited from eight communes in the rural district of Cam Lam, Khanh Hoa province, in Central Vietnam. Self-reported eating speed was assessed on a 5-point Likert scale, and responses were collapsed into the following three categories: slow, normal, and fast. Abdominal obesity was defined as a waist-to-height ratio of ≥ 0.5. Poisson regression with a robust variance estimator was used to assess the association between eating speed and abdominal obesity.

**Results:**

Compared with slow eating speed, the adjusted prevalence ratio (95% confidence interval) for abdominal obesity was 1.14 (1.05, 1.25)1.14 (1.05, 1.25) for normal eating speed and 1.30 (1.19, 1.41) for fast eating speed (P for trend < 0.001).

**Conclusion:**

A faster eating speed was associated with a higher prevalence of abdominal obesity in a middle-aged population in rural Vietnam.

**Supplementary Information:**

The online version contains supplementary material available at 10.1186/s12889-023-15328-0.

## Background

Obesity continues to be a major public health concern. Approximately 1.9 billion adults worldwide were estimated to be overweight in 2016, with 650 million being affected by obesity, the prevalence of which has tripled over the past four decades [1]. Obesity is an important risk factor for various chronic diseases, [1] including diabetes, cardiovascular disease, [2] and certain types of cancer [3]. A chronic excess of energy intake over expenditure is the fundamental cause of obesity, [1] and this positive energy imbalance usually results from the combination of overeating and physical inactivity, both of which are modifiable in terms of obesity prevention [4, 5]

Epidemiological data show that fast eating speed may be a modifiable risk factor for obesity. A meta-analysis of 23 cross-sectional and prospective studies reported fast eating speed was associated with both a higher body mass index [BMI], which is the most common index of overall adiposity, and a greater risk of general obesity defined by BMI [6]. Specifically, the pooled odds ratio for general obesity (BMI ≥ 25 kg/m^2^) was 2.15 (95% confidence interval [CI]: 1.84, 2.51) comparing the fastest vs. slowest eating. This suggests that controlling eating speed can be a possible approach for regulating body weight and preventing obesity.

Although abdominal obesity may pose a higher threat to health than general obesity, particularly in Asian populations, [7–10] there remains some issues pertaining to the association between eating speed and abdominal obesity have not yet been investigated. Of the three common measurements of abdominal obesity, i.e., waist circumference (WC), waist-to-hip ratio and waist-to-height ratio (WHtR), the latter appears to be the best predictor for cardiometabolic risk; a WHtR of ≥ 0.5 has been widely used to define abdominal obesity, for both sex [11–13]. However, the current data on the relationship between eating speed and WHtR are derived from children populations [14–16]. In adults, there is evidence for the association between eating speed and WC, but no evidence for the association between eating speed and WHtR [17, 18]. In addition, the majority of the previous studies on the association between eating speed and adiposity are from high-income countries, while little is known about this issue in low- and middle-income countries, which are currently undergoing a rapid nutrition transition [19] and experiencing a drastic increase in obesity prevalence [20]. The identification of dietary behaviors associated with abdominal adiposity may facilitate efforts to reduce the disease burden, particularly in low-resource settings.

Therefore, the present study aimed to examine the association between eating speed and abdominal obesity (defined as WHtR ≥ 0.5) in a Vietnamese middle-aged population. We hypothesized that a faster eating speed would be associated with a higher prevalence of abdominal adiposity.

## Methods

### Study setting

The present data were a part of the Khanh Hoa Cardiovascular Study which is an ongoing cohort study on the determinants of cardiovascular disease among the Vietnamese population. The study sites consist of eight communes in the rural district of Cam Lam, Khanh Hoa province, in Central Vietnam, which are deemed average in terms of their affluence in rural Vietnam. The participants were recruited among commune residents who were between the ages of 40 and 59 years at the time of recruitment. All eligible residents, who were listed by local commune health center staff members, were invited until we reached a sample size of 3000 participants (consent rate: 75–87%).

The baseline survey, which collected information on lifestyle parameters, anthropometry, and biochemical measurements, was conducted between June 2019 and June 2020 among the 3000 participants. Face-to-face interviews and anthropometric measurements were carried out at selected commune health centers, and biochemical measurements were performed at the Pasteur Institute in Nha Trang, Khanh Hoa. It should be mentioned that the sample size was not calculated specifically to examine the association between eating speed and abdominal obesity.

### Anthropometric measurements

WC was measured to the nearest 0.1 cm (at the umbilical level) using a measuring tape, with the participant in the standing position. The WHtR was obtained by dividing the WC (cm) by the height (cm). Body height and weight were measured to the nearest 0.1 cm and 0.1 kg, respectively, using a digital scale (Tanita, HD-661, Tokyo, Japan) and a portable stadiometer (Charder, HM200P, Tokyo, Japan). All measurements were performed by trained staff members, and the same measurement protocol was used throughout the study period.

### Adiposity

The main outcome was abdominal obesity, which was defined as a WHtR of ≥ 0.50 [11–13]. For the purpose of comparison, we also considered two secondary outcomes: high waist circumference (WC of ≥ 90 cm in men or ≥ 80 cm in women) and general obesity (defined as BMI [calculated as weight in kilograms divided by height in meters squared] ≥ 25 kg/m^2^) according to the criteria of the World Health Organization for the Asian population [21].

### Eating speed

Eating speed was assessed with the following question: “how would you describe your speed of eating?”. The five response options were “very slow,” “relatively slow,” “normal,” “relatively quick,” and “very quick.” Due to the very low frequency of the first and last responses (n = 39, and n = 100, respectively), we regrouped the responses into three new categories: slow (very slow and relatively slow), normal, and fast (relatively quick and very quick) eating speeds.

### Covariates

We collected information pertaining to the following socio-demographic covariates via a questionnaire: age (years), sex (men or women), marital status (married or not married), education level (less than primary school, primary school, secondary school, or high school or higher), job category (government employees, non-government employees, self-employed, farmers or fishermen, housewives, others, or unemployed), and household income (low, middle, or high). The household income per month (in Vietnamese dong; 23,475 dong were equivalent to 1 United States dollar as of June 1, 2019) was estimated by the household representative and categorized into the following levels: ≤1,000,000; 1,000,001 to ≤ 2,000,000; 2,000,001 to ≤ 3,000,000; 3,000,001 to ≤ 4,000,000; 4,000,001 to ≤ 6,000,000; 6,000,001 to ≤ 8,000,000; 8,000,001 to ≤ 12,000,000; 12,000,001 to ≤ 16,000,000; 16,000,001 to ≤ 20,000,000; >20,000,000; or do not know). Each response was assigned the midpoint of the range as a proxy score. The values were divided by the square root of the number of household members to obtain the equivalized income, which was then categorized into tertiles.

Other covariates included smoking (never, former, or current smoker); alcohol consumption (non-drinker or drinker consuming < 1, 1–1.9, or ≥ 2 standard drinks/day); sleeping hours (< 6; 6–6.9, 7–7.9, 8–8.9, or ≥ 9 h/day); addition of sugar to beverages (yes or no); consumption of soft drinks (yes or no), fruits/vegetables (< 1, 1–1.99, 2–2.99, 3–2.99, 4–4.99, or ≥ 5 servings/day), rice (< 3, 3–5, 6–8, or ≥ 8 bowls/day), and meat (< 100, 100–199, 200–299, or ≥ 300 g/day); and a medical history (yes or no) of cancer, cardiovascular disease, or antidiabetic medication use (yes or no). Physical activity (total metabolic equivalent task) was assessed using the Global Physical Activity Questionnaire, [22] and scores were categorized into tertiles (low, middle, or high).

### Statistical analysis

Participant characteristics are presented as mean (standard deviation [SD]) or median (interquartile range [IQR]) for continuous variables, and percentage for categorical variables. A Poisson regression model with a robust variance estimator was used to estimate the prevalence ratio (PR) and 95% CI of abdominal obesity in relation to eating speed. To account for missing data on household income (i.e., “do not know”; n = 33), we used multiple imputation to create 20 datasets [23] and combined them according to Rubin’s rule [24]. The main analyses consisted of two models. Model 1: unadjusted; Model 2: adjusted for age, sex, commune, education, marital status, occupation, household income, smoking, alcohol, physical activity, sleeping hours, adding sugar to beverages, medical history of cancer or diseases of the circulatory system, and using antidiabetic medication. Subsequently, the analyses were stratified by sex to determine if the relationship between eating speed and abdominal obesity would be different between men and women. We also stratified the analyses by smoking status, and alcohol consumption (among men only [n = 1160], since almost all female participants did not smoke tobacco [99.3%], or drink alcohol [97.3%]). The trend association between eating speed and abdominal obesity was assessed by assigning an ordinal number (1 to 3) to the eating speed category (slow, normal, and fast), which was then treated as a continuous variable in the regression models. We also assessed the association between eating speed and the WHtR which was treated as a continuous variable in linear regression models.

To test the robustness of the study findings, we conducted a series of sensitivity analyses. First, we excluded those with missing information on household income (n = 33). Second, we excluded those with medical history of cancer, cardiovascular diseases, or antidiabetic medication use (n = 154) to rule out potential effect of these factors. Finally, we examined the association of eating speed with high waist circumference, and general obesity.

Statistical significance was set at P < 0.05. All statistical analyses were performed using RStudio (version 3.2.4, RStudio Team, Boston, USA) [25].

## Results

Among 3,000 participants, 1,073 (35.8%) and 502 (16.7%) reported that they were fast eaters and slow eaters, respectively (Table 1). Participants who were fast eaters tended to be men, overweight, governmental employees, and ex- or current smokers. They were also more likely to drink a higher amount of alcohol, add sugar to beverages, and eat more rice and meat than participants who were slow eaters.

**Table 1:** Basic characteristics of study participants, 2019–2020

Characteristics	All participants (n = 3000)	Eating speed			
		Slow (n = 502)	Normal (n = 1425)	Fast (n = 1073)	
Age, mean [standard deviation]	48.7 [5.5]	49.3 [5.8]	48.8 [5.5]	48.3 [5.4]	
Sex (men)	1160 (38.7)	154 (30.7)	535 (37.5)	471 (43.9)	
Body mass index					
18.5-	139 (4.6)	40 (8.0)	69 (4.8)	30 (2.8)	
18.5–24.9	2083 (69.4)	364 (72.5)	1008 (70.7)	711 (66.3)	
≥ 25	778 (25.9)	98 (19.5)	348 (24.4)	332 (30.9)	
Education					
Primary school	352 (11.7)	60 (12.0)	169 (11.9)	123 (11.5)	
Secondary school	863 (28.8)	126 (25.1)	416 (29.2)	321 (29.9)	
High school	1068 (35.6)	184 (36.7)	495 (34.7)	389 (36.3)	
Tertiary study	717 (23.9)	132 (26.3)	345 (24.2)	240 (22.4)	
Married	2691 (89.7)	427 (85.1)	1278 (89.7)	986 (91.9)	
Occupation					
Government employee	295 (9.8)	43 (8.6)	137 (9.6)	115 (10.7)	
Non-government employee	483 (16.1)	80 (15.9)	212 (14.9)	191 (17.8)	
Self-employed	595 (19.8)	103 (20.5)	263 (18.5)	229 (21.3)	
Farmer/fisherman	870 (29.0)	141 (28.1)	415 (29.1)	314 (29.3)	
Others	757 (25.2)	135 (26.9)	398 (27.9)	224 (20.9)	
Household income					
Low tertile	1087 (36.2)	181 (36.1)	543 (38.1)	363 (33.8)	
Middle tertile	930 (31.0)	154 (30.7)	439 (30.8)	337 (31.4)	
High tertile	983 (32.8)	167 (33.3)	443 (31.1)	373 (34.8)	
Smoking					
Non-smoker	2036 (67.9)	365 (72.7)	1000 (70.2)	671 (62.5)	
Ex-smoker	350 (11.7)	57 (11.4)	140 (9.8)	153 (14.3)	
Current smoker	614 (20.5)	80 (15.9)	285 (20.0)	249 (23.2)	
Alcohol consumption					
Non-drinker	2114 (70.5)	381 (75.9)	1039 (72.9)	694 (64.7)	
Drinker consuming					
< 1 standard drink/day	416 (13.9)	59 (11.8)	187 (13.1)	170 (15.8)	
1-1.9 standard drink/day	201 (6.7)	25 (5.0)	99 (6.9)	77 (7.2)	
≥ 2 standard drink/day	269 (9.0)	37 (7.4)	100 (7.0)	132 (12.3)	
Physical activity (MET-h/week)					
Low	1030 (34.3)	142 (28.3)	565 (39.6)	323 (30.1)	
Middle	975 (32.5)	170 (33.9)	463 (32.5)	342 (31.9)	
High	995 (33.2)	190 (37.8)	397 (27.9)	408 (38.0)	
Sleeping hours per day					
< 6	485 (16.2)	77 (15.3)	227 (15.9)	181 (16.9)	
6-6.9	669 (22.3)	123 (24.5)	318 (22.3)	228 (21.2)	
7-7.9	915 (30.5)	147 (29.3)	441 (30.9)	327 (30.5)	
8-8.9	613 (20.4)	112 (22.3)	294 (20.6)	207 (19.3)	
≥ 9	318 (10.6)	43 (8.6)	145 (10.3)	130 (12.1)	
Adding sugar to beverages (yes)	1088 (36.3)	177 (35.3)	469 (32.9)	442 (41.2)	
Consumption of soft drink (yes)	454 (15.1)	85 (16.9)	198 (13.9)	171 (15.9)	
Fruit/vegetable [serving/day] ^†^	2.0 [1.9]	2.0 [1.8]	1.7 [1.4]	2.0 [1.8]	
Rice consumption [bowl/day] ^†^	4.0 [3.0]	4.0 [4.0]	4.0 [3.0]	4.0 [3.0]	
Meat consumption [gram/day] ^†^	82.9 [100.0]	85.0 [100.0]	80.0 [94.3]	85.7 [122.9]	
Using antidiabetic medication (yes)	93 (3.1)	13 (2.6)	47 (3.3)	33 (3.1)	
History of diseases of the circulatory system (yes)	51 (1.7)	9 (1.8)	27 (1.9)	15 (1.4)	
History of cancer (yes)	18 (0.6)	2 (0.4)	8 (0.6)	8 (0.7)	

Figures are n (%), unless otherwise stated; ^†^ values are median [interquartile range]; MET: metabolic equivalent task

Using the WHtR, 63.6% of participants were identified as having an abdominal obesity. High waist circumference and general obesity were identified in 39.0% and 25.9% of the participants, respectively. A significant association was found between eating speed and abdominal obesity (Table 2). The crude PR (95% CI) for abdominal obesity associated with normal and fast eating speeds were 1.14 (1.05, 1.25) and 1.27 (1.16, 1.38), respectively (P for trend < 0.001), compared with slow eating speed. The associations remained significant after an adjustment for all confounders: the adjusted PR (95% CI) were 1.14 (1.05, 1.25) and 1.30 (1.19, 1.41) for normal and fast eating speeds, respectively (P for trend < 0.001).

**Table 2:** Association between eating speed and abdominal obesity among 3000 participants of the baseline survey of the Khanh Hoa Cardiovascular Study in Vietnam (2019–2020)

Eating speed	Abdominal obesity^†^ n (%)	Prevalence ratio (95% confidence interval)	
		Model 1	Model 2
**Overall**			
Slow	275 (54.8)	1.00 (ref)	1.00 (ref)
Normal	889 (62.4)	1.14 (1.05, 1.25)	1.14 (1.05, 1.25)
Fast	744 (69.3)	1.27 (1.16, 1.38)	1.30 (1.19, 1.41)
*P trend*		*< 0.001*	*< 0.001*
**Men**			
Slow	71 (10.8)	1.00 (ref)	1.00 (ref)
Normal	287 (43.8)	1.16 (0.96, 1.40)	1.18 (0.99, 1.41)
Fast	297 (45.3)	1.37 (1.14, 1.64)	1.41 (1.18, 1.68)
*P trend*		*0.007*	*0.009*
**Women**			
Slow	204 (16.3)	1.00 (ref)	1.00 (ref)
Normal	602 (48.0)	1.15 (1.04, 1.27)	1.16 (1.05, 1.27)
Fast	447 (35.7)	1.27 (1.15, 1.40)	1.26 (1.15, 1.39)
*P trend*		*0.005*	*0.008*

^†^ waist-to-heigh ratio ≥ 0.5; ref: reference; Model 1: unadjusted; Model 2: adjusted for age, commune, education, marital status, occupation, household income, smoking, alcohol, physical activity, sleeping hours, adding sugar to beverages, medical history of cancer or diseases of the circulatory system, and using antidiabetic medication

When we stratified the analysis by sex (Table 2), smoking status (Table S1), and alcohol consumption (Table S2), we did not observe pronounced differences in the associations between eating speed and abdominal obesity among the subgroups. Those who self-reported to eat fast tended to be abdominally obese than those who self-reported to eat slowly.

The sensitivity analysis after excluding of participants with missing information on household income resulted in similar results (e.g., the adjusted PR [95% CI] for abdominal obesity was 1.29 [1.19, 1.41], comparing fast versus slow eating speeds [Table 3]). Similarly, the exclusion of history of cancer, cardiovascular diseases, or antidiabetic medication use also did not materially change the associations between eating speed and abdominal obesity (Table 4). Eating speed was also associated with the continuous values of WHtR, e.g., adjusted coefficient for fast vs. slow eating speed was 0.017 (95% CI: 0.012, 0.023) (Table S3).

**Table 3:** Association between eating speed and abdominal obesity, excluding participants with missing information on household income (n = 33)

Eating speed	Abdominal obesity^†^ n (%)	Prevalence ratio (95% confidence interval)	
		Model 1	Model 2
Slow	271 (55.0)	1.00 (ref)	1.00 (ref)
Normal	880 (62.4)	1.14 (1.04, 1.24)	1.14 (1.04, 1.24)
Fast	739 (69.5)	1.26 (1.16, 1.38)	1.29 (1.19, 1.41)
*P trend*		*< 0.001*	*< 0.001*

^†^waist-to-heigh ratio ≥ 0.5; ref: reference; Model 1: unadjusted; Model 2: adjusted for age, sex, commune, education, marital status, occupation, household income, smoking, alcohol, physical activity, sleeping hours, adding sugar to beverages, medical history of cancer or diseases of the circulatory system, and using antidiabetic medication

**Table 4:** Association between eating speed and abdominal obesity, excluding participants with medical history of cancer or diseases of the circulatory system, or using antidiabetic medication (n = 153)

Eating speed	Abdominal obesity^†^ n (%)	Prevalence ratio (95% confidence interval)	
		Model 1	Model 2
Slow	262 (54.7)	1.00 (ref)	1.00 (ref)
Normal	825 (61.2)	1.12 (1.02, 1.23)	1.13 (1.03, 1.23)
Fast	702 (68.8)	1.26 (1.15, 1.38)	1.29 (1.18, 1.41)
*P trend*		*< 0.001*	*< 0.001*

^†^waist-to-heigh ratio ≥ 0.5; ref: reference; Model 1: unadjusted; Model 2: adjusted for age, sex, commune, education, marital status, occupation, household income, smoking, alcohol, physical activity, sleeping hours, and adding sugar to beverages

A similar pattern of associations was also observed for high waist circumference (Table S4) and general obesity defined with BMI (Table S5). For example, the adjusted prevalence ratios (95% CI) of high waist circumference and general obesity were 1.41 (1.23, 1.61; P for trend < 0.001) and 1.51 (1.24, 1.84; P for trend < 0.001), respectively, for the comparison between fast and slow eating speeds.

## Discussion

In the present study, we found that fast eating speed was significantly associated with a higher prevalence of abdominal obesity, as defined according to the WHtR, among a rural middle-aged population in Vietnam. To the best of our knowledge, no prior studies have investigated the relationship between eating speed and WHtR in adults.

Our findings are supported by existing epidemiological data on the association of fast eating speed with high WC [17, 18] and visceral fat accumulation [26, 27]. For example, in a meta-analysis of 11 studies on metabolic syndrome, [18] fast eating speed was associated with high WC (odds ratio: 1.54; 95% CI: 1.37, 1.73), which is a component of metabolic syndrome [28]. In a Japanese cross-sectional study, [26] fast eating speed was associated with a higher odds of an increased visceral fat area (i.e., ≥ 100 cm2, as determined via computed tomography scans) (odds ratio: 1.99; 95% CI: 1.40, 2.90). Our results provide additional evidence to support the assertion that fast eating speed is associated with abdominal obesity, which may pose a greater health risk than general obesity [7–10].

Although the mechanism by which fast eating speed affects abdominal adiposity remains unclear, it is possible that fast eating speed may lead to excessive caloric intake, which in turn results in abdominal obesity. The over-intake of calories may be related to a delay in the onset of satiation and the feeling of fullness. Fast eating speed may reduce the secretion of peptide YY [29] (a postprandial hormone that induces satiety), [30] thereby delaying the sensation of fullness, which results in the overconsumption of food. Studies have shown that fast eating speed can lead to increased energy intake [31] which is a major driver of obesity [4]. For example, in a Singaporean cohort study, [32] fast eaters consumed 105 kcal/day more than slow eaters. In the present study, fast eaters appeared to consume more rice and meat than slow eaters (Table 1). Furthermore, fast eating speed results in a shorter oral processing time, which may also contribute to the delayed onset of satiation and increased food intake [33, 34]

In addition to the overconsumption of food, heavy congestion of dietary fat also may partly explain. During the postprandial stage, an excessive intake of dietary fat leads to a congestion of free fatty acids in the intestinal lamina propria, which is where free fatty acids are directly transported to the abdominal visceral adipocytes [35]. The heavier congestion results in the delivery of more free fatty acids and subsequent accumulation in the abdominal visceral adipocytes [36]. This may partly explain our observed association between fast eating speed and abdominal adiposity.

In the present study, fast eating speed was associated with both abdominal and general adiposity; however, the former association may be more informative for public health strategies aimed at mitigating obesity-related health risks. Compared with general obesity, abdominal obesity is a stronger predictor of cardiovascular diseases, [8] cancer, [9] and mortality [10]. The detrimental impact of abdominal obesity on health risk can be even significant among normal-weight people [7]. Given the high global prevalence of individuals with normal-weight abdominal obesity (e.g., 19.9% in Japanese adults [37] and 29.5% in South African adults [38]), the identification of preventable risk factors of abdominal fat accumulation is crucial to establish appropriate strategies for mitigating obesity-related health risks. The present finding suggests that preventive measures for abdominal adiposity should account for the role of eating speed.

The results of the present study should be interpreted with several considerations. First, the eating speed was not objectively measured, but self-reported by participants, which might have affected the accuracy of estimates. Nevertheless, this asssessment method has been frequently utilized in previous studies on this topic [6]. Second, our brief dietary questionnaire did not allow us to calculate the total energy intake, which did not allow us to estimate the possible role of caloric intake in the association between eating speed and abdominal obesity. It is possible that those who eat fast tend to consume more calories or that those who eat a lot need to eat fast. Third, given the cross-sectional design of this study, we were unable to infer a causal relationship between fast eating speed and abdominal obesity. Finally, since our study population comprised middle-aged rural residents in a Central province of Vietnam, the generalization of the study findings to other population groups should be made with caution.

## Conclusion

The present study found that a faster eating speed was associated with a higher prevalence of abdominal obesity among a middle-aged population in rural Vietnam.

## Electronic supplementary material

Below is the link to the electronic supplementary material.

Supplementary Material 1

## Data Availability

The datasets analyzed during the current study are not publicly available due the ethical requirements of the research proposal but are available from the National Center for Global Health and Medicine on reasonable request.
