# Supplementary material for: Eating speed and abdominal adiposity in middle-aged adults: a cross-sectional study in Vietnam
Source: BMC Public Health. 2023 Mar 7;23:443. doi: 10.1186/s12889-023-15328-0 (PMC9993665; doi:10.1186/s12889-023-15328-0)
Supplement: Supplementary file 1 — Supplementary Material 1 [file 12889_2023_15328_MOESM1_ESM.docx]

**Eating speed and abdominal adiposity in mid-aged adults: a cross-sectional study in Vietnam**

Table S1. Association between eating speed and abdominal obesity by smoking status among 1160 male participants of the baseline survey of the Khanh Hoa Cardiovascular Study, Vietnam (2019-2020)

| Eating speed | Abdominal obesity^†^  n (%) | Prevalence ratio (95% confidence interval) | |
| --- | --- | --- | --- |
|  |  | Model 1 | Model 2 |
| Non-smoker | | | |
| Slow | 8 (42.1) | 1.00 (ref) | 1.00 (ref) |
| Normal | 82 (70.1) | 1.58 (0.94, 2.65) | 1.44 (0.88, 2.38) |
| Fast | 52 (71.2) | 1.67 (0.98, 2.84) | 1.56 (0.94, 2.60) |
| P trend |  | 0.31 | 0.26 |
| Ex-smoker | | | |
| Slow | 33 (58.9) | 1.00 (ref) | 1.00 (ref) |
| Normal | 79 (57.7) | 1.00 (0.77, 1.30) | 1.04 (0.79, 1.37) |
| Fast | 106 (69.7) | 1.25 (0.97, 1.61) | 1.22 (0.94, 1.59) |
| P trend |  | 0.24 | 0.18 |
| Current smoker | | | |
| Slow | 30 (38.0) | 1.00 (ref) | 1.00 (ref) |
| Normal | 126 (44.8) | 1.20 (0.90, 1.59) | 1.18 (0.88, 1.58) |
| Fast | 139 (56.5) | 1.48 (1.11, 1.96) | 1.41 (1.06, 1.89) |
| P trend |  | 0.018 | 0.031 |
| ^†^ waist-to-heigh ratio ≥ 0.5; ref: reference; Model 1: unadjusted; Model 2: adjusted for age, commune, education, marital status, occupation, household income, alcohol, physical activity, sleeping hours, adding sugar to beverages, medical history of cancer or diseases of the circulatory system, and using antidiabetic medication. | | | |

Table S2. Association between eating speed and abdominal obesity by alcohol consumption levels among 1160 male participants of the baseline survey of the Khanh Hoa Cardiovascular Study, Vietnam (2019-2020)

| Eating speed | Abdominal obesity,^†^ n (%) | Prevalence ratio (95% confidence interval) | |
| --- | --- | --- | --- |
|  |  | Model 1 | Model 2 |
| Non-drinker | | | |
| Slow | 20 (45.5) | 1.00 (ref) | 1.00 (ref) |
| Normal | 95 (57.2) | 1.17 (0.85, 1.62) | 1.22 (0.87, 1.72) |
| Fast | 64 (56.1) | 1.28 (0.92, 1.80) | 1.29 (0.91, 1.83) |
| P trend |  | 0.55 | 0.34 |
| Drinkers consuming <1 standard drink | | | |
| Slow | 23 (46.0) | 1.00 (ref) | 1.00 (ref) |
| Normal | 83 (48.8) | 1.09 (0.79, 1.51) | 1.13 (0.82, 1.57) |
| Fast | 95 (62.9) | 1.37 (1.00, 1.89) | 1.42 (1.03, 1.95) |
| P trend |  | 0.08 | 0.11 |
| Drinkers consuming <1-2 standard drinks | | | |
| Slow | 9 (39.1) | 1.00 (ref) | 1.00 (ref) |
| Normal | 56 (56.6) | 1.46 (0.90, 2.37) | 1.7 (1.03, 2.81) |
| Fast | 51 (68.9) | 1.83 (1.13, 2.97) | 1.92 (1.17, 3.16) |
| P trend |  | 0.09 | 0.08 |
| Drinkers consuming ≥2 standard drinks | | | |
| Slow | 19 (51.4) | 1.00 (ref) | 1.00 (ref) |
| Normal | 53 (53.0) | 1.13 (0.80, 1.60) | 1.11 (0.77, 1.58) |
| Fast | 87 (65.9) | 1.29 (0.93, 1.80) | 1.29 (0.92, 1.80) |
| P trend |  | 0.19 | 0.26 |
| ^†^ waist-to-heigh ratio ≥ 0.5; ref: reference; Model 1: unadjusted; Model 2: adjusted for age, commune, education, marital status, occupation, household income, alcohol, physical activity, sleeping hours, adding sugar to beverages, medical history of cancer or diseases of the circulatory system, and using antidiabetic medication. | | | |

Table S3. Association between eating speed and waist-to-height ratio among 3000 participants of the baseline survey of the Khanh Hoa Cardiovascular Study in Vietnam (2019-2020)

| **Eating speed** | **N** | **Waist-to-heigh ratio**  **mean [SD]** | **Coefficient (95% confidence interval)** | |
| --- | --- | --- | --- | --- |
|  |  |  | Model 1 | Model 2 |
| Slow | 502 | 0.511 [0.057] | 0.00 (ref) | 0.00 (ref) |
| Normal | 1425 | 0.518 [0.052] | 0.008 (0.002, 0.013) | 0.008 (0.003, 0.013) |
| Fast | 1073 | 0.526 [0.053] | 0.015 (0.009, 0.021) | 0.017 (0.012, 0.023) |
| P trend |  |  | < 0.001 | < 0.001 |
| SD: standard deviation; ref: reference; Model 1: unadjusted; Model 2: adjusted for age, sex, commune, education, marital status, occupation, household income, alcohol, physical activity, sleeping hours, adding sugar to beverages, medical history of cancer or diseases of the circulatory system, and using antidiabetic medication. | | | | |

# Table S4. Association between eating speed and high waist circumference among 3000 participants of the baseline survey of the Khanh Hoa Cardiovascular Study in Vietnam (2019-2020)

| **Eating speed** | **High waist circumference^†^**  **N (%)** | **Prevalence ratio (95% confidence interval)** | |
| --- | --- | --- | --- |
|  |  | Model 1 | Model 2 |
| Slow | 169 (33.7) | 1.00 (ref) | 1.00 (ref) |
| Normal | 533 (37.4) | 1.11 (0.97, 1.28) | 1.16 (1.01, 1.32) |
| Fast | 469 (43.7) | 1.30 (1.13, 1.49) | 1.41 (1.23, 1.61) |
| *P trend* |  | *< 0.001* | *< 0.001* |
| **^†^** waist circumference [WC] ≥ 90 cm in men or WC ≥ 80 cm in women; ref: reference; Model 1: unadjusted; Model 2: adjusted for age, sex, commune, education, marital status, occupation, household income, alcohol, physical activity, sleeping hours, adding sugar to beverages, medical history of cancer or diseases of the circulatory system, and using antidiabetic medication. | | | |

# Table S5. Association between eating speed and general obesity among 3000 participants of the baseline survey of the Khanh Hoa Cardiovascular Study in Vietnam (2019-2020)

| **Eating speed** | **General obesity ^†^**  **N (%)** | **Prevalence ratio (95% confidence interval)** | |
| --- | --- | --- | --- |
|  |  | Model 1 | Model 2 |
| Slow | 98 (19.5) | 1.00 (ref) | 1.00 (ref) |
| Normal | 348 (24.4) | 1.25 (1.02, 1.53) | 1.23 (1.01, 1.49) |
| Fast | 332 (30.9) | 1.58 (1.30, 1.93) | 1.51 (1.24, 1.84) |
| P trend |  | < 0.001 | < 0.001 |
| **^†^** body mass index ≥ 25 kg/m^2^; ref: reference; Model 1: unadjusted; Model 2: Model 2: adjusted for age, sex, commune, education, marital status, occupation, household income, alcohol, physical activity, sleeping hours, adding sugar to beverages, medical history of cancer or diseases of the circulatory system, and using antidiabetic medication. | | | |
